# Supplementary material for: Optical nanomanipulation on solid substrates via optothermally-gated photon nudging
Source: Nat Commun. 2019 Dec 12;10:5672. doi: 10.1038/s41467-019-13676-3 (PMC6908671; doi:10.1038/s41467-019-13676-3)
Supplement: Supplementary file 1 — Supplementary Information [file 41467_2019_13676_MOESM1_ESM.pdf]

Supplementary Information

"Optical nanomanipulation on solid substrates via optothermally-gated photon nudging"

Li et al.

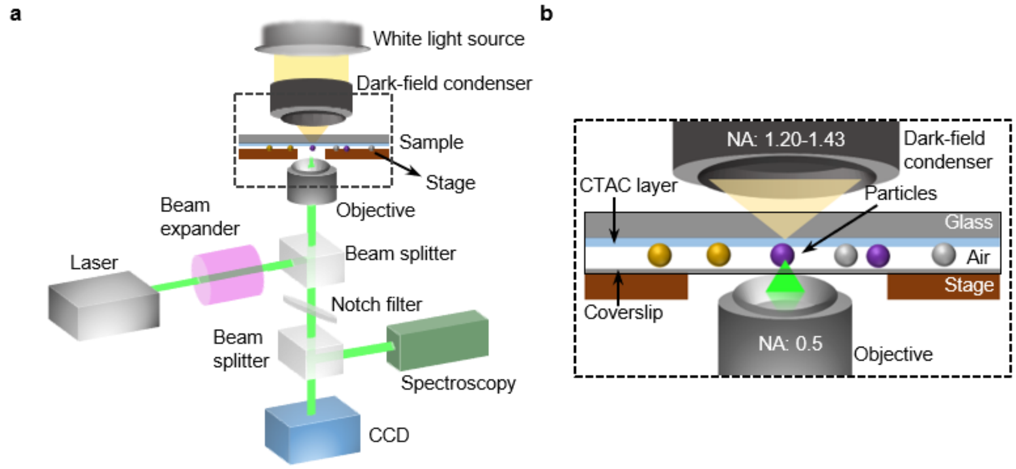

Supplementary Figure 1. Experimental setup for OPN. a, The optical setup for OPN and in situ spectroscopy. **b**, Detailed setup of sample configuration and dark-field scattering measurement.

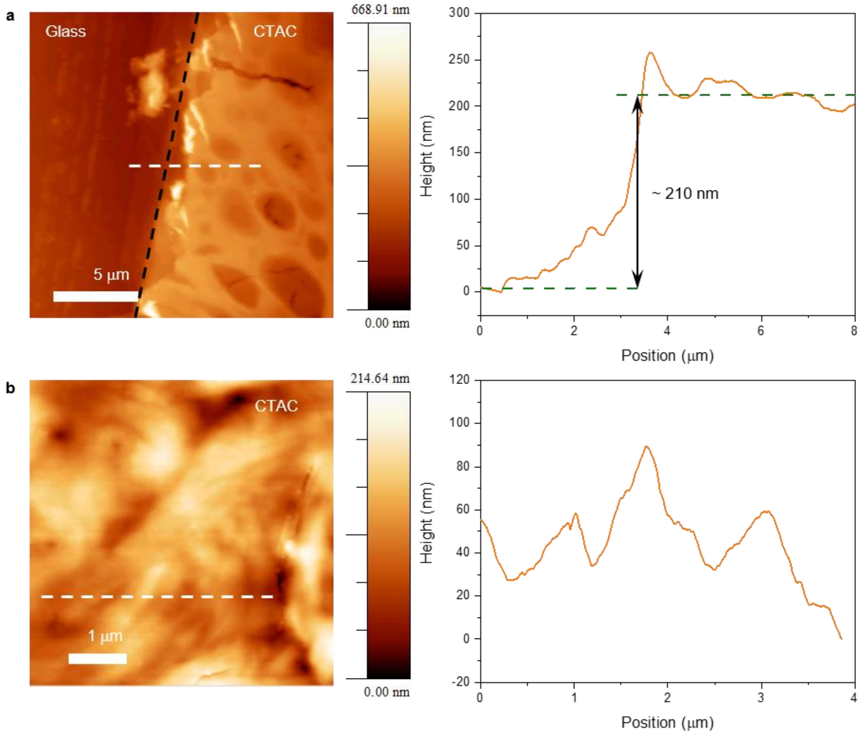

Supplementary Figure 2. Surface topography characterization of CTAC layer. AFM images and the corresponding height profiles of the white dashed lines: **a,** the boundary between CTAC layer and the glass

substrate (marked with the black dashed line); **b**, the CTAC layer. The root mean square (RMS) roughness in (**b**) is ~32 nm.

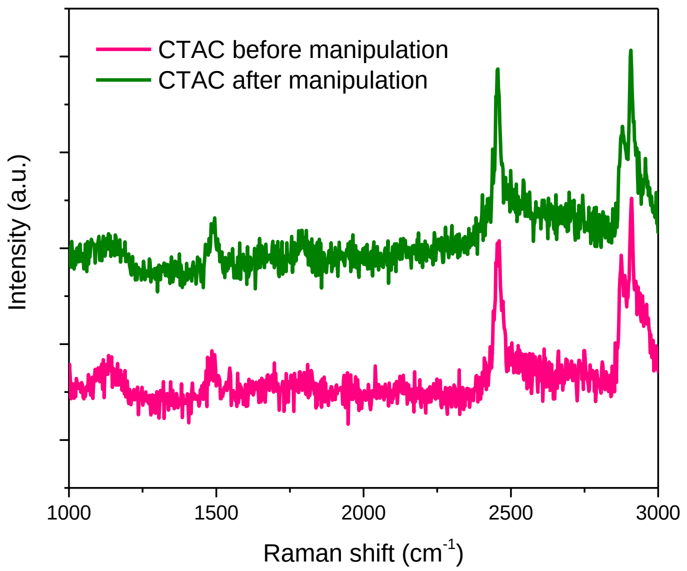

Supplementary Figure 3. Raman Spectra of CTAC before and after OPN manipulation. The Raman spectra were measured using the in situ spectroscopy in OPN. Before manipulation, the Raman spectrum is consistent with the reported Raman spectrum of CTAC powders[1]. This result further confirmed that CTAC forms a thin solid layer at room temperature before optical heating. After we manipulated the colloidal particles, we measured the signal again at the area where the particle passed through. No obvious changes were observed in the Raman spectra. These results suggest that CTAC can well recover to its solid phase after the particle manipulation, which can continuously serve as the optothermal gate for OPN.

Supplementary Table 1. Summary of the control experiments to investigate the role of optical heating and scattering forces in OPN.

No.	Particle	Substrate	CTAC?	Optical heating	Optical scattering	OPN works or not?
**1**	Au/Ag/Si	Glass	No	Strong	Strong	No
**2**	Au/Ag/Si	Glass	Yes	Strong	Strong	**Yes**
**3**	PS	Glass	Yes	Weak	Weak	No
**4**	PS	AuNIs	Yes	Strong	Weak	No
**5**	TiO_2	Glass	Yes	Weak	Strong	No
**6**	TiO_2	AuNIs	Yes	Strong	Strong	**Yes**

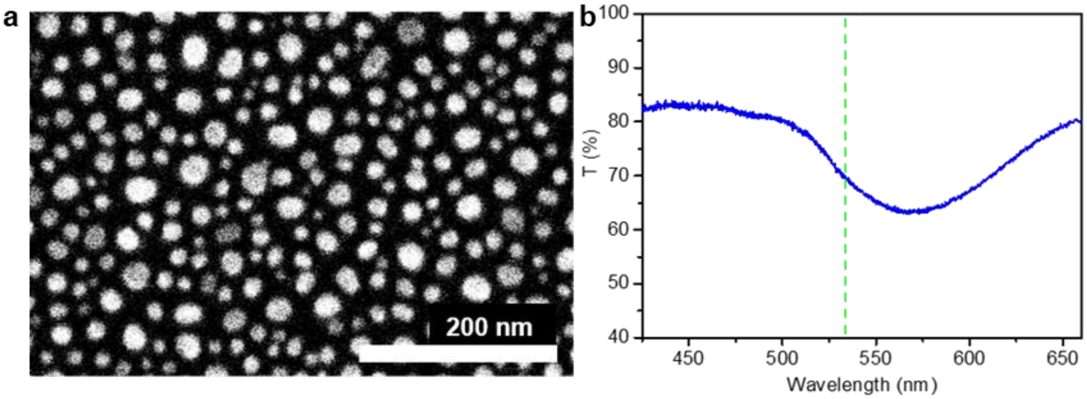

Supplementary Figure 4. Characterization of the thermoplasmonic substrtae. a, Scanning electron micrograph (SEM) of AuNIs substrate after thermal annealing of a 4.5 nm (in thickness) Au thin film. **b**, The corresponding transmission spectra. The green dashed line indicates the wavelength of the laser (532 nm).

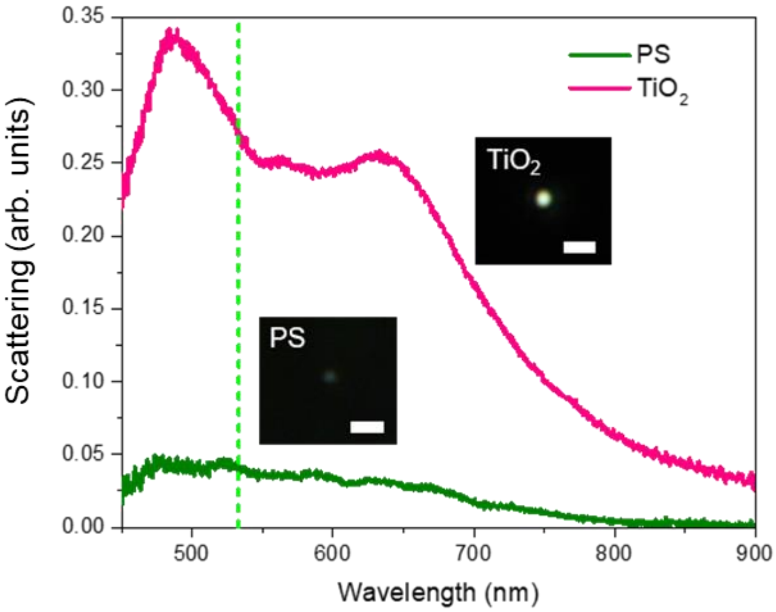

Supplementary Figure 5. Scattering spectra of TiO_2 nanoparticle (pink) and PS (olive) measured at the same incident light intensity. The insets show the corresponding dark-filed images, scale bars: 2μm. The green dashed line indicates the wavelength of the laser (532 nm). Apparently, PS nanoparticles show weak scattering at the laser wavelength, while TiO_2 nanoparticles have a much larger scattering efficiency.

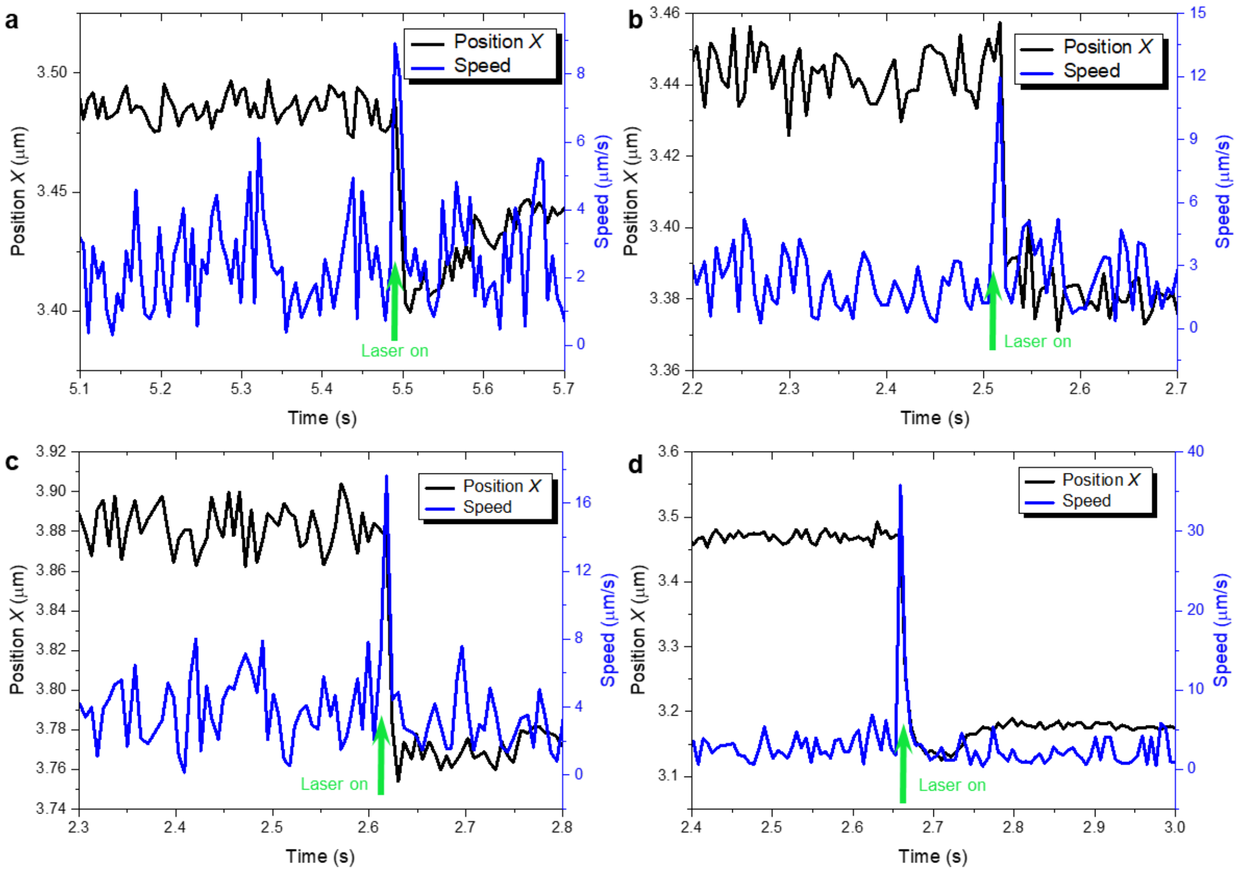

Supplementary Figure 6. Measured position, speed, and acceleration of a 300 nm AuNP. Incident power: **a**, 0.27 mW; **b**, 0.58 mW; **c**, 0.96 mW; **d**, 1.40 mW. The green arrows indicate the instants when the laser was turned on. The acceleration values showed in the figure corresponded to the accelerations when the laser was on, which were directly extracted from the software. With an increasing optical power, the particle gained both larger speed and great acceleration.

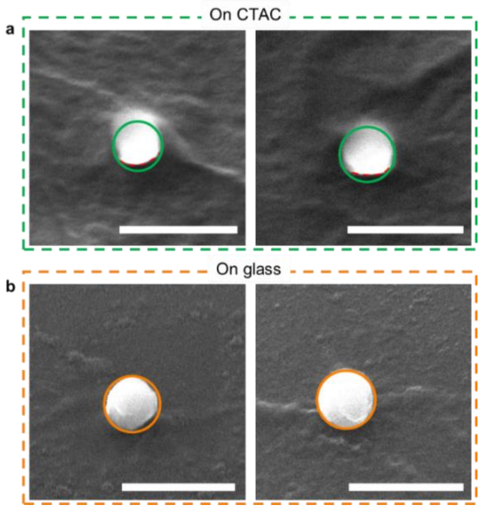

Supplementary Figure 7. 45° titled SEM images of 200 nm AuNPs. a, AuNPs on a CTAC film; **b**, AuNPs on a glass substrate. There is an obvious immersion line for the AuNPs on a CTAC film (red dashed lines in **a**), while for the glass substrate, the AuNPs maintains its spherical shape. These results clearly show that the particles are partially immersed into CTAC film during OPN manipulation.

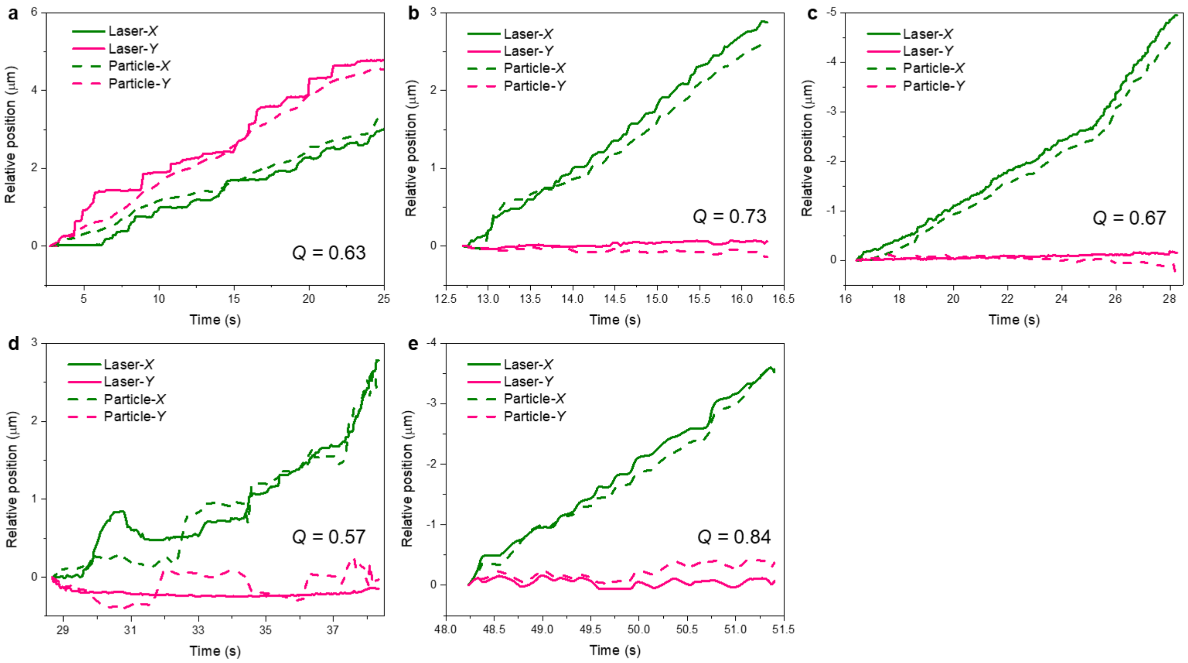

Supplementary Figure 8. Trajectories of the particles and laser beam recorded in Supplementary Movie 1. a, 200 nm AuNP; **b**, 80 nm AuNP, moving forth; **c**, 80 nm AuNP, moving back; **d**, 100 nm AgNP; **e**, 500 nm SiNP. The manipulation efficiencies of each particle are also presented in the figures.

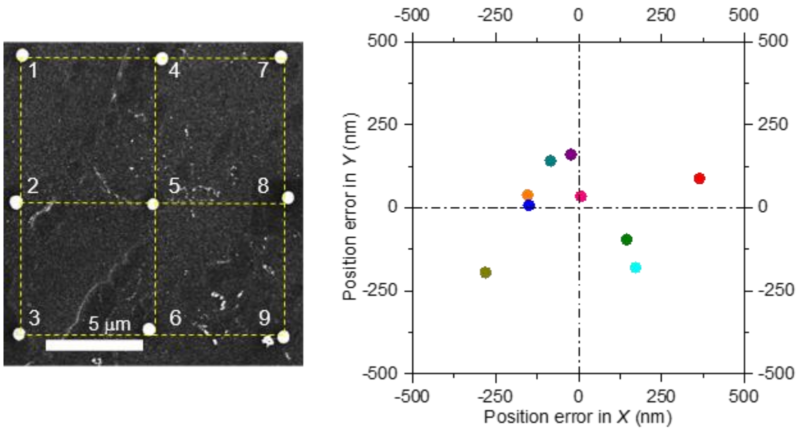

Supplementary Figure 9. Detailed information for the position errors of SiNPs in the 2D 3×3 array. All particles are numbered as indicated in the SEM image. The determination of position errors in *X* and *Y* directions can be found in Fig. 3i.

Supplementary Table 2. Detailed information for the position errors of each SiNPs in the 2D 3×3 array. The particle numbers are consistent with those in Supplementary Fig. 9.

Particle No.	Symbol	Position error in X (nm)	Position error in Y (nm)	Absolute position error (nm)
1	●	145.6	-95.8	174.3
2	●	-153.2	38.3	158.0
3	●	7.7	34.5	35.3
4	●	364.0	88.1	374.5
5	●	-84.3	141.8	164.9
6	●	-279.7	-195.4	341.2
7	●	-149.4	7.7	149.6
8	●	172.4	-180.1	249.3
9	●	-23.0	160.9	162.5

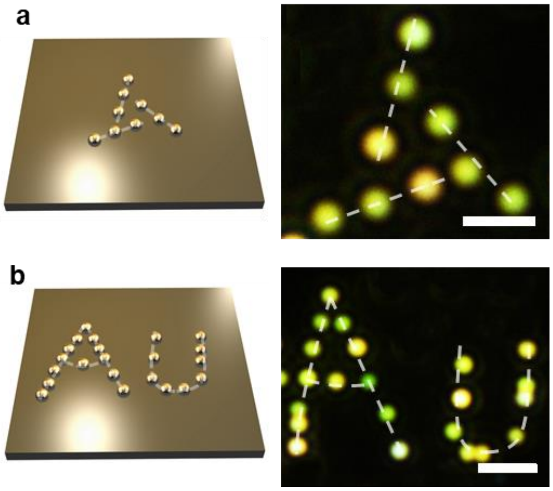

Supplementary Figure 10. Patterning of 80 nm AuNPs into (**a**) a triangular spiral composed of 9 particles and (**b**) a “Au” pattern composed of 22 particles. Scale bars: **a**, 2 µm; b, **5** µm.

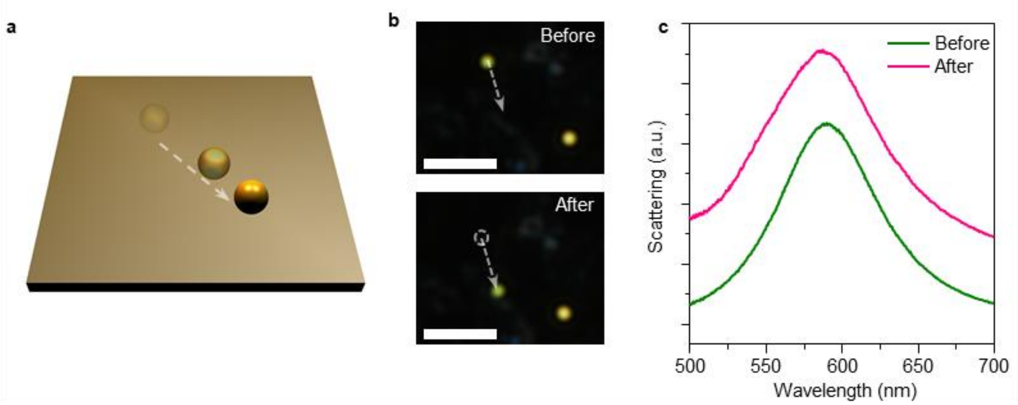

Supplementary Figure 11. a, Schematic illustration of the AuNP manipulation process. **b,c,** Dark-field images and scattering spectra of the AuNP before and after OPN manipulation. Scale bars: 5 µm. The scattering spectra showed no apparent differences after the OPN manipulation, which indicates OPN can manipulate nanoparticles without damaging their optical properties.

Supplementary Note 1. Understanding the role of optical heating and scattering forces

In order to understand the role of optical heating and scattering, we designed and conducted a series of control experiments. AuNPs (or AgNPs, SiNPs) are known to exhbit strong light absorption and light scatterning[2,3]. To decouple the optical heating and scattering forces and explore the effects of each factor, we applied two strategies. First, we used a thermoplasmonic substrate composed of quasicontinous gold nanoislands (AuNIs) as the heat source (Supplementary Fig. 4). Localized surface plasmon resonances were excited on the AuNIs substrate under the radiation of a 532 nm laser beam with both high-efficiency light absorption and photon-phonon conversion to generate well-confined and localized thermal hotspots[4]. With this setup, AuNIs can effectively serve as external heating sources, which allows us to explore the manipulation of particles with weak photothermal responses. Second, we used Polysterene (PS) and titanium dioxide (TiO_2) nanoparticles for the control experiments. Both PS and TiO_2 nanoparticles had negligible optical absorption at 532 nm[5,6]. While TiO_2 nanoparticles showed strong light scattering at this wavelength and PS nanoparticles showed much weaker scattering (Supplementary Fig. 5).

The parameters and results of the control experiments are summarized in Supplementary Table 1. In the absence of the CTAC layer, all particles were firmly bonded onto the glass substrate through strong van der Waals interaction[7] and could not be moved by the laser beam. By adding the CTAC layer, Au (or Ag and Si) nanoparticles were able to be manipulated with both optical heating and scattering forces using OPN (Supplementary Movie 1). Since PS and TiO_2 nanoparticles have weak optical absorption, they could not be manipulated without AuNIs, which highlights the importance of optical heating. This crucial optical heating caused by the AuNIs opened the CTAC optothermal gate, allowing the TiO_2 nanoparticles to be manipulated by optical scattering forces (Supplementary Movie 2). Contrastingly, PS nanoparticles could not be manipulated even under high-power radiation due to the lack of light scattering, its driving force. These results

undoubtedly show that OPN simultaneously exploits optical heating to open the optothermal gate and radiation-pressure forces to drive the particles.

Supplementary Note 2. Excluding the thermal expansion force and electrostatic force in OPN as the driving forces

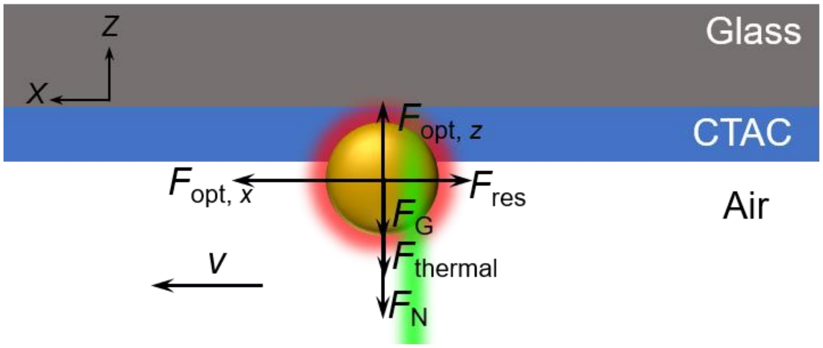

Supplementary Figure 12. The scheme of force analysis in OPN platform under laser irradiation. In the *Z*-direction, there is a gravity force $\mathbf{F}_{G}$, an optical force $\mathbf{F}_{opt,\,z}$, a normal force $\mathbf{F}_{N}$, and a thermal expansion force $\mathbf{F}_{thermal}$. Since the particles are moved along the *X*-*Y* plane, all forces in the *Z*-direction are balanced. In the *X*-direction, there is an optical force $\mathbf{F}_{opt,\,x}$ and a resistance force $\mathbf{F}_{res}$. Both particles and the CTAC layer are charged, there are also electrostatic forces $\mathbf{F}_{electro}$ which are not depicted in the schematic. The forces shown in the scheme are not scaled.

Multiple forces exist in this OPN platform, including optical, thermal expansion, electrostatic, and resistance forces (Supplementary Fig. 12). We excluded the thermal expansion and electrostatic force as the primary driving forces in OPN. In our experimental setup (Supplementary Fig. 1), the thermal expansion force imposes a downward momentum to the particle and drives the particle away from the CTAC layer[8]. By directing a laser beam with an increased optical power of 2.7 mW at a 200 nm AuNP, we observed the immediate launch

of the AuNP to another position (Supplementary Movie 3). The fact that the AuNP still remained on the substrate indicates that the out-plane thermal expansion force is not dominant, and the AuNP was quickly pushed to the new position by the in-plane optical force instead.

Since both particles and CTAC layers are charged, there are electrostatic forces in the OPN system. We replaced the cationic CTAC with an anionic sufactant, sodium dodecyl sulfate (SDS)[9], to rule out the electrostatic force. Particles on a SDS layer can be manipulated with the laser beam similar to those on a CTAC layer (Supplementary Movie 4), confirming the minor role of eletrostatic force in OPN.

Supplementary Note 3. Calculating resistant forces in OPN

During OPN manipulation, colloidal particles are partially immersed into the quasi-liquid CTAC film (Supplementary Fig. 7). Thus, the resistance force to the colloidal particle during the manipulation can be modelled as[10, 11]

$$\mathbf{F}_{\mathrm{res}}=6\pi\eta R f_{\mathrm{d}}\mathbf{v} \quad (1)$$

where η is the viscosity of the quasi-liquid CTAC, R is the radius of the particle, f_{d} is a dimensionless drag coefficient, and $\boldsymbol{v}$ is the velocity of the particle. To calculate $\mathbf{F}_{\mathrm{res}}$, we determined η and f_{d} as discussed below.

3.1 MD simulation of the viscosity of the quasi-liquid CTAC

The viscosity of CTAC in its quasi-liquid state was calculated by molecular dynamics (MD) simulations with the LAMMPS package[12]. The velocity Verlet algorithm is employed in integrating equations of motion, and the time step is 0.25 fs. Initially, the isothermal–isobaric (NPT) ensemble is employed to reach the required temperature and pressure (1 atm). Then, the system is equilibrated under the canonical ensemble (NVT) with the Langevin heat reservoir at the target temperature for 0.5 ps, followed by relaxation under a microcanonical ensemble (NVE) for 0.2 ns. Finally, a production step of 10 ns was adopted under the NVE condition, during

which the pressure tensor was calculated every 10 fs to obtain PACF. For each case, four independent simulations were performed with different initial atom velocity assignments, implemented by using different seeds for random number generation. Averaged values were obtained to improve the reliability of the simulation results. The SHAKE algorithm was employed to fix geometries of the water molecules and partial bonds of CTAC molecule[13]. Long-range electrostatic interactions were counted using the particle-particle particlemesh method[14] with a precision of 10^{-6}.

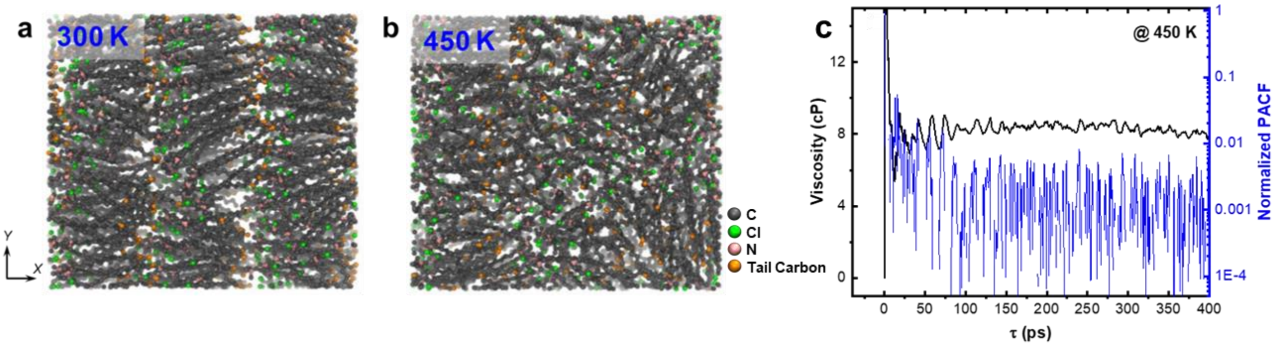

Supplementary Figure 13. MD simulation of CTAC. a,**b**, Snapshots of CTAC in MD simulations at (**a**) 300 K and (**b**) 450 K. **c**, Calculated viscosity and normalized pressure tensor autocorrection function (PACF) of CTAC at 450 K.

During the OPN manipulation, the temperature of CTAC surrounding the AuNP exceeds 450 K (Fig. 1d). Thus, we simulated the structure and viscosity of CTAC at 450 K. An order-disorder phase transition of CTAC can clearly be observed when temperature increases from 298 K to 450 K (Supplementary Fig. 13a,b), confirming the formation of quasi-liquid CTAC. The result is consistent with the reported publications[15]. The viscosity of the quasi-liquid CTAC is calculated based on Green-Kubo linear response theory by the integral of pressure tensor autocorrelation function (PACF) via[16]

$$\eta = \frac{\mathrm{V}}{k_{\mathrm{B}}\mathrm{T}} \int_{o}^{\tau} \langle P_{\alpha\beta}(0) P_{\alpha\beta}(t) \rangle dt \qquad (2)$$

where V is the system volume, k_B is the Boltzmann constant, T is the temperature and τ is correlation time. The angle bracket denotes time correlation function, also interpreted as ensemble averaging. $P_{\alpha\beta}$ is an off-diagonal ($\alpha, \beta = x, y, z;\ \alpha \neq \beta$) element of the pressure tensor, which for an N-particle system is calculated by

$$P_{\alpha\beta} = \frac{1}{V}\sum_{i=1}^{N}\left(m_i v_{i\alpha} v_{i\beta} + r_{i\alpha} f_{i\beta}\right) \qquad (3)$$

where m_i, v_i, r_i and f_i are the mass, velocity, position, and force of the atom i, respectively. The simulated viscosity at 450 K was calculated to be 8.7 mPa·s (Supplementary Fig. 13c). Which was used for calculation of the resistant forces.

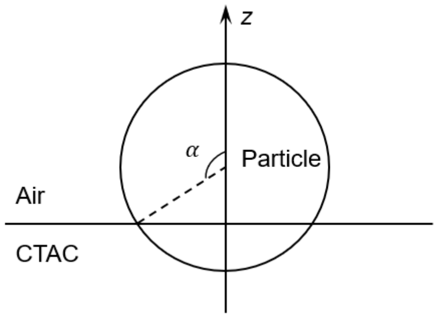

Supplementary Figure 14. Geometry of our system to define the contact angle α.

3.2 Determination of the drag coefficient

According to the theory developed by Danov *et al*[11], when a colloidal particle is half-immersed in a thin liquid film, the drag coefficient f_d is only a function of the contact angle α (Supplementary Fig. 14) and the surface viscosity. In our system, CTAC has a free surface (no liquid-liquid interface), therefore, the surface viscosity = 0. In this case, f_d is almost independent of the contact angle[11]. When α = 90°, f_d equals 0.5 corresponding to the half-immersed case. In our case, the contact angle is larger than 90° (Supplementary Fig. 7), and the corresponding f_d is smaller than 0.5. Based on the results by Danov *et al*[11], the drag coefficient in our case was estimated to be ~0.3-0.4 for the calculation of resistant forces. As an example, we calculated the resistant

force for 200 nm AuNPs based on the experimental velocities. At the optical power of 1.4 mW, the measured velocity is ~70 μm s^{-1}, and the calculated resistant force is ~0.35 pN, which is in similar range of the optical forces.

Supplementary Note 4. Improving patterning accuracy with the assistance of the imaging software

The patterning accuracy of OPN is primarily limited by the optical diffraction limit. Additionally, we only relied on our naked eyes to estimate the positions of the nanoparticles during the experiments. Here, we demonstrate that it is possible to apply the predesigned markers or imaging software to define the target lines for particle manipulation. As shown in Supplementary Fig. 15a, we used the imaging software to define the target line and moved five SiNPs to the lines. With the assistance of the target lines, the average position error was reduced to less than ~30 nm (Supplementary Fig. 15b,c).

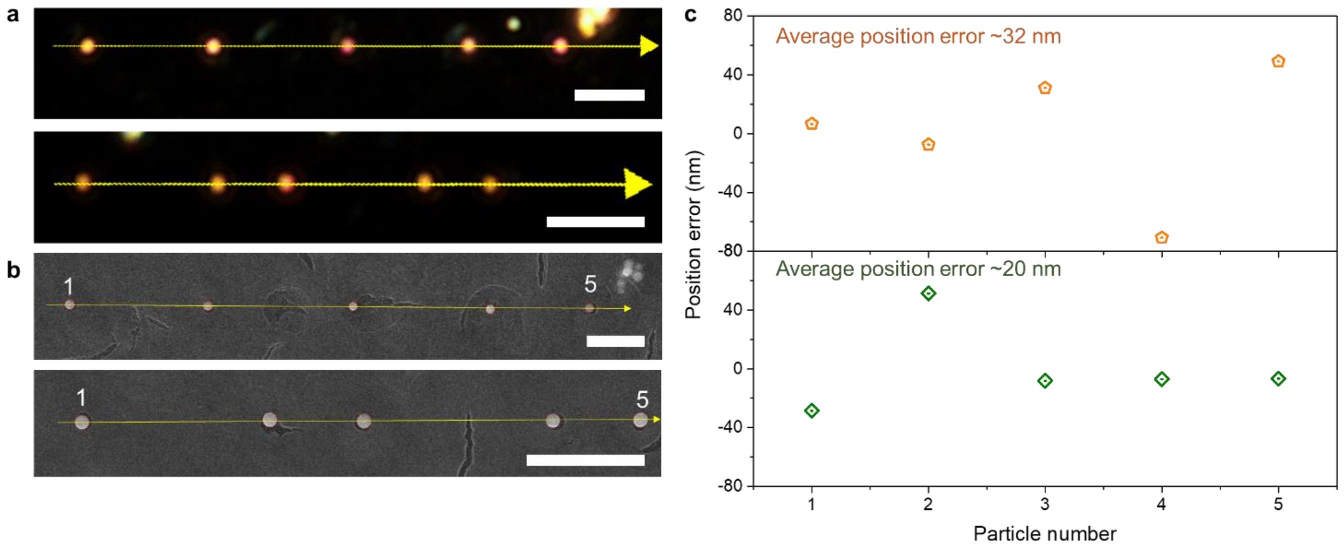

Supplementary Figure 15. Precise patterning of 500 nm SiNPs with the the assistance of the software. **a**,**b,** Dark-field images (**a**) and SEM images (**b**) of two patterned lines of 5 SiNPs. **c**, The corresponding position errors of each particles in (**b**). All Scale bars are 5 μm.

Supplementary Note 5. In situ spectroscopy to distinguish the colloidal particles with different sizes

We applied the in situ spectroscopy to measure the experimental spectra to verify the different sizes of the particles. As shown in Supplementary Fig. 16, the colour of the AuNPs changes from green to yellow when the diameter increases from 40 to 100 nm. The corresponding scattering peak redshifts from 545 to 590 nm. Similarly, when the size of AgNPs increased from 80 nm to 110 nm, the hue of the particles changes from blue to cyan, with a redshift of the scattering peak position from 465 to 480 nm. Since we do not need the laser to trap the target colloidal particle during the spectral measurement, our OPN can effectively avoid the interference from the laser beam. For instance, while using a 532 nm laser, we can still record the scattering spectra at that specific wavelength (Supplementary Fig. 16).

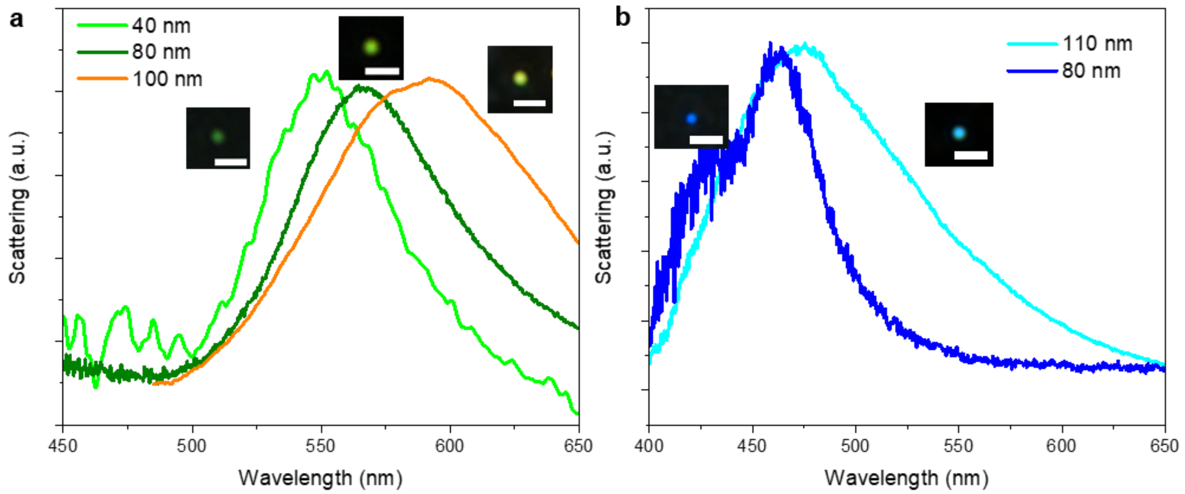

Supplementary Figure 16. In situ Scattering spectra of AuNPs and AgNPs. a, Scattering spectra of (green) 40 nm, (olive) 80 nm, and (orange) 100 nm AuNPs. **b**, Scattering spectrum of (blue) 80 nm and (cyan)110 nm AgNPs. The insets show the corresponding dark-field images. All scale bars are 2 μm.

Supplementary Note 6. Reliable fabrication of Au dimers with ~15 nm gap

The diffraction limit in optical microscopic imaging has prevented the measurement of the distance between two close nanoparticles. In this experiment, two 100 nm AuNPs (Supplementary Fig. 17d) were assembled into a Au dimer. Supplementary Fig. 17a,b show the dark-field optical images of the Au dimer composed of two same 100 nm AuNPs with different interparticle distances. It is challenging to distinguish the gap between these two AuNPs based on the optical images. However, taking advantage of our in situ optical spectroscopy, one can easily obtain the scattering spectra of the Au dimers. As shown in Supplementary Fig. 17e, the scattering spectrum of the dimer in Supplementary Fig. 17a shows a single peak at ~585 nm, which is consistent with that of single AuNPs. The spectrum can also be well-fitted by a single-peak Lorentz function. The result reveals that there is no near-field coupling between two AuNPs, which indicates two AuNPs are separated by a large distance. In contrast, the dimer in Supplementary Fig. 17b shows two split peaks at ~575 nm and ~635 nm (Supplementary Fig. 17e), which demonstrate the near-field coupling behavior between these two AuNPs. The peak splitting results are consistent with those shown in Fig. 5. The SEM image further confirmed a Au dimer with a gap of ~ 15 nm (Supplementary Fig. 17c). In summary, we can reliably fabricate the Au dimer with the desired gap by analysing the scattering spectra with the help of in situ optical spectroscopy. This strategy is generally applicable to all types of nanoparticles and substrates. However, the effects of orientations of anisotropic nanoparticles need be considered in establishing the relationship between the interparticle gaps and scattering spectra. The in situ optical spectroscopy that can monitor the scattering spectra of colloidal structures and estimate the interparticle gaps during the assembly process will significantly benefit the investigation of light-matter interactions and coupling of colloidal particles.

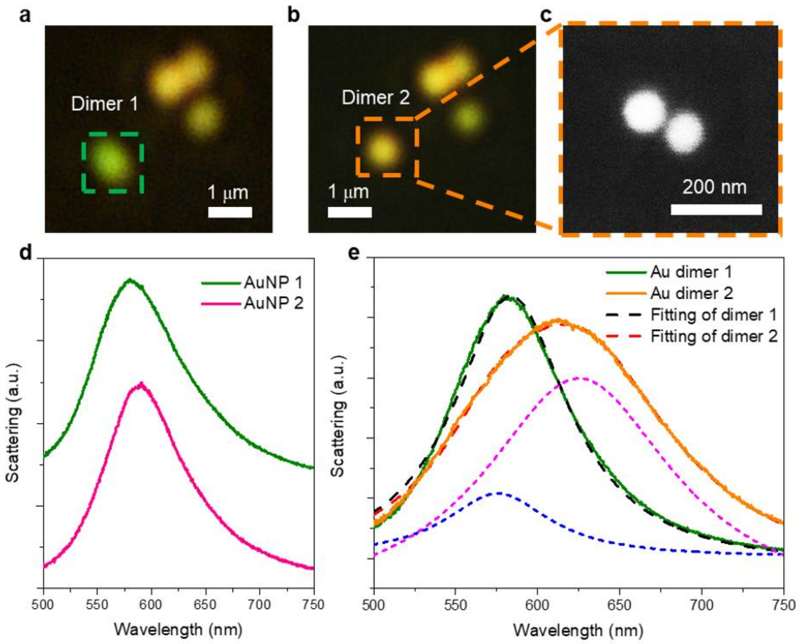

Supplementary Figure 17. Reliable fabrication of Au dimer with a gap of ~15 nm. a,**b**, Optical images of the Au dimer composed of two same 100 nm AuNPs with different interparticle distances. **c**, The SEM images of the Au dimer in (**b**). **d**, The scattering spectra of the two single AuNPs. **e**, Scattering spectra and the fitting of the Au dimers in (**a**) and (**b**).

Supplementary Note 7. Optimal wavelength for OPN

The working wavelengths for OPN can be properly optimized to reduce the optical power needed to open the optothermal gate and improve the performance. Take the manipulation of 80 nm AuNPs as an example, in which both 532 nm and 660 nm lasers were tested for OPN experiment. The results showed that the 80 nm AuNPs can be readily manipulated by 532 nm laser with a low power of 1.0 mW. In contrast, 660 nm laser doesn't work even with a much higher power of 2.5 mW. This is because 80 nm AuNPs have much stronger absorption at 532 nm than that at 660 nm (Supplementary Fig. 18c). The simulated temperature also shows that the temperature surrounding the particle reaches more than 450 K for the 532 nm laser, opening the optothermal gate, while a 660 nm laser produces a temperature of only ~330 K (Supplementary Fig. 18a,b). Therefore, the optimal working wavelengths for OPN can be selected to match the absorption cross-section of the colloidal particles. In addition, as we demonstrated, it is also possible to introduce other optothermal materials (such as gold nanoislands) as external heating sources to induce the phase transition of CTAC, thus, the optimal wavelength, in this case, should be chosen based on the absorption of the external optothermal materials to open the optothermal gate.

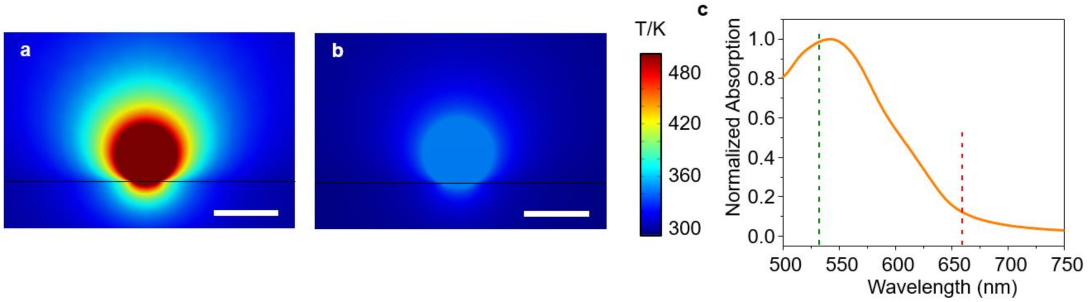

Supplementary Figure 18. Optimal working wavelength for OPN. a,**b**, Simulated temperature distribution around an 80 nm AuNP. (**a**) 532 nm laser with an incident power of 1 mW; (**b**) 660 nm laser with an incident power of 2.5 mW. Scale bars: 80 nm. **c**, Simulated absorption cross-section for 80 nm AuNPs. The green and red dashed lines stand for the wavelengths of two lasers used.

Supplementary References

1. Li, X. et al. Pd-on-NiCu nanosheets with enhanced electro-catalytic performances for methanol oxidation. *J. Alloys Compd.* **685**, 42-49 (2016).
2. Baffou, G. & Quidant, R. Thermo-plasmonics: Using metallic nanostructures as nano-sources of heat. *Laser Photonics Rev.* **7**, 171-187 (2013).
3. Fu, Y. H. et al. Directional visible light scattering by silicon nanoparticles. *Nat. Commun.* **4**, 1527 (2013).
4. Lin, L. H. et al. Optothermoplasmonic nanolithography for on-demand patterning of 2D materials. *Adv. Funct. Mater.* **28**, 1803990 (2018).
5. Zhao, Y. et al. Synthesis and optical properties of TiO_2 nanoparticles. *Mater. Lett.* **61**, 79-83 (2007).
6. Li, T., Zhou, C. L. & Jiang, M. UV absorption-spectra of polystyrene. *Polym. Bull.* **25**, 211-216 (1991).
7. Urban, A. S., Lutich, A. A., Stefani, F. D. & Feldmann, J. Laser printing single gold nanoparticles. *Nano Lett.* **10**, 4794-4798 (2010).
8. Alam, M. S. & Zhao, C. Nondestructive approach for additive nanomanufacturing of metallic nanostructures in the air. *ACS Omega* **3**, 1213-1219 (2018).
9. Casson, B. D. & Bain, C. D. Phase transitions in mixed monolayers of sodium dodecyl sulfate and dodecanol at the air/water interface. *J. Phys. Chem. B* **102**, 7434-7441 (1998).
10. Kralchevsky, P. A. & Nagayama, K. Capillary interactions between particles bound to interfaces, liquid films and biomembranes. *Adv. Colloid. Interfaces* **85**, 145-192 (2000).
11. Danov, K., Aust, R., Durst, F. & Lange, U. Influence of the surface viscosity on the hydrodynamic resistance and surface diffusivity of a large brownian particle. *J. Colloid. Interface Sci.* **175**, 36-45 (1995).

12. Plimpton, S. Fast parallel algorithms for short-range molecular dynamics. *J. Comput. Phys.* **117**, 1-19 (1995).

13. Hockney, R. W. & Eastwood, J. W. *Computer simulation using particles*. (CRC Press, 1988).

14. Sun, H. COMPASS: An ab initio force-field optimized for condensed-phase applications overview with details on alkane and benzene compounds. *J. Phys. Chem. B* **102**, 7338-7364 (1998).

15. Tian, Y. C. et al. Impedance spectroscopic study on rotator and disordered phases in trimethylammonium chlorides. *J. Phys. Chem. C* **120**, 23905-23909 (2016).

16. Allen, M. P. & Tildesley, D. J. *Computer simulation of liquids*. (Oxford University Press, 2017).
