# Supplementary material for: Optical nanomanipulation on solid substrates via optothermally-gated photon nudging
Source: Nat Commun. 2019 Dec 12;10:5672. doi: 10.1038/s41467-019-13676-3 (PMC6908671; doi:10.1038/s41467-019-13676-3)
Supplement: Supplementary file 2 — Description of Additional Supplementary Files [file 41467_2019_13676_MOESM2_ESM.pdf]

Description of Additional Supplementary Files

Supplementary Movie 1: Nanomanipulation of diverse colloidal particles with different sizes.

Supplementary Movie 2: Manipulation of TiO_2 nanoparticles on CTAC layer with and without AuNIs substrate.

Supplementary Movie 3: In-plane launch of a 200 nm AuNP with an optical power of 2.7 mW.

Supplementary Movie 4: Manipulation of a 200 nm AuNP on a thin layer of SDS.

Supplementary Movie 5: Power-dependent velocity measurement for 300 nm AuNPs. The white “+” indicates the original positions of AuNPs.

Supplementary Movie 6: Dynamic rotation and translation of AuNWs.
